# Abnormalities in aortic arch geometry do not lead to reduced exercise performance: a comparison study between patients with transposition of the great arteries repaired by arterial switch operation and normal controls

**DOI:** 10.1186/1532-429X-15-S1-P291

**Published:** 2013-01-30

**Authors:** Hopewell Ntsinjana, Giovanni Biglino, Claudio Capelli, Alessandro Giardini, Graham Derrick, Silvia Schievano, Andrew M Taylor

**Affiliations:** 1 Centre for Cardiovascular Imaging, Institute of Cardiovascular Science, University College London, London, UK; 2 Cardioespiratory Unit, Great Ormond Street Hospital for Children NHS Foundation Trust, London, UK

## Background

Abnormal aortic arch geometry, in particular the ‘gothic arch', has been associated with reduced exercise performance in patients with repaired aortic coarctation. Following the arterial switch operation (ASO) for transposition of the great arteries (TGA), the morphology of the aorta is also known to be abnormal, presenting with dilated aortic root and ‘gothic' deformity of the aortic arch. In this study, our aim was to compare aortic geometry between normal and ASO patients to establish if aortic arch shape influenced exercise performance.

## Methods

**Figure 1:**
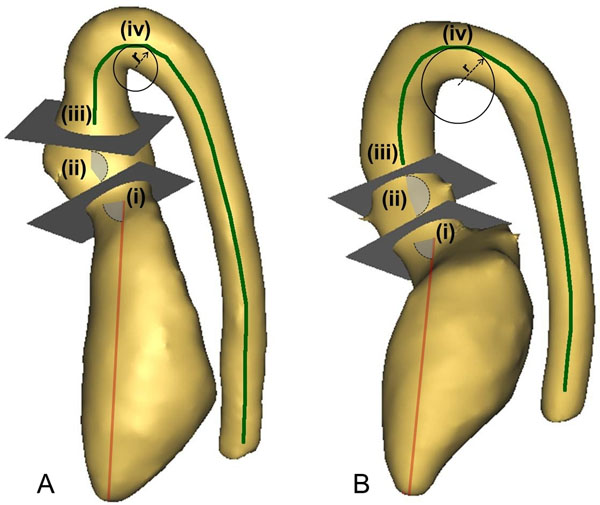
Identification of geometrical markers and measures on 3D models of a TGA subject (A) and of a control case (B), as described in the Methods section.

We studied 17 TGA patients repaired by ASO (age=15.3±1.8 years, BSA=1.8±0.2 m^2^) and 17 matched controls (age=14.9±1.9 years, BSA=1.6±0.2 m^2^). All subjects signed informed consent. 3D b-SSFP whole heart CMR images were acquired for 3D volume reconstruction. 3D anatomical models of the left heart including LV, LVOT, aortic root, ascending aorta, aortic arch and descending aorta to the level of diaphragm were created using commercial software (Mimics). Geometrical analysis (Figure 1) was performed measuring: (i) the angle between the line connecting the centre of the valve with the apex of the LV and the aortic valve plane; (ii) the angle between the aortic valve plane and the sinotubular junction plane; (iii) the indexed length of the vessel centreline starting from the centre of the aortic valve to the level of the diaphragm; (iv) the indexed curvature of the aortic arch, from the inverse of the radius (r) of the maximum circumference fitted at the highest point of the centreline (=1/r). On the same day as the CMR scan, cardiopulmonary exercise test was performed in all subjects using an electronically braked ergometer cycle. Exercise capacity, heart rate and systolic blood pressure response to exercise were measured.

## Results

There were significant differences in aortic geometry between patients and controls (Table 1). However, there are no notable differences in physiological response to exercise between the two groups.

**Table 1:** Differences in geometric variables and hemodynamic response to exercise between TGA subjects and controls. p<0.05 is considered statistically significant.

Variable	TGA Subjects	Controls	p-value
LV-AV angle [°]	62 ± 9	63 ± 8	0.65
AV-STJ angle [°]	151 ± 10	172 ± 3	<0.001
Aortic centreline length [mm/m^2^]	140 ± 22	123 ± 12	0.008
Aortic arch curviture [1/mm/m^2^]	0.053 ± 0.016	0.020 ± 0.004	<0.001
VO_2_ max [mL/kg/min]	39.4 ± 5.0	36.6 ± 7.8	0.23
Peak blood pressure [mmHg]	151 ± 20	152 ± 21	0.96
Peak heart rate [bpm]	175 ± 10	175 ± 13	0.99

## Conclusions

Using ASO as a model for alteration in aortic arch geometry without direct operation on the arch, we show that marked changes in aortic shape are not associated with changes in exercise capacity, raising the possibility that the ‘gothic arch' appearances seen in aortic coarctation do not play a role in the exercise hypertensive response seen in such coarctation patients.

## Funding

Commonwealth Scholarships; National Institute of Health Research UK (NIHR); Fondation Leducq; Royal Academy of Engineering and EPSRC

